# Epidemic ribotypes of *Clostridium (*now *Clostridioides) difficile* are likely to be more virulent than non-epidemic ribotypes in animal models

**DOI:** 10.1186/s12866-020-1710-5

**Published:** 2020-02-05

**Authors:** John C. Vitucci, Mark Pulse, Leslie Tabor-Simecka, Jerry Simecka

**Affiliations:** 1 Department of Pharmaceutical Sciences and UNTHSC Preclinical Services, University of North Texas System College of Pharmacy, University of North Texas Health Science Center, Fort Worth, TX USA; 2 Department of Research, Reata Pharmaceuticals, Irving, TX USA

**Keywords:** *Clostridium*, *Clostridioides*, *Difficile*, Animal models, Virulence, In vitro phenotype, Ribotype, Epidemic, Toxin

## Abstract

**Background:**

*Clostridioides difficile* infections have become more frequently diagnosed and associated with greater disease severity, which has resulted in an increase burden on the healthcare system. These increases are attributed to the increased prevalence of hypervirulent strains encompassing select ribotypes. These epidemic ribotypes were characterized as hypervirulent due to higher in vitro spore and toxin production, as well as increased incidence, severity and mortality within patients. However, it is unclear whether epidemic ribotypes are truly more virulent than non-epidemic ribotypes in vivo*.* Furthermore, there is conflicting evidence about the ability of a strain’s in vitro phenotype to be predictive of their in vivo virulence. The goals of the current studies were to determine if epidemic ribotypes are more virulent than other ribotypes in animal models, and whether the in vitro virulence phenotype of an isolate or ribotype predict in vivo virulence.

**Results:**

To determine if epidemic strains were truly more virulent than other non-epidemic strains, the in vivo virulence of 13 *C. difficile* isolates (7 non-epidemic and 6 epidemic ribotype isolates) were determined in murine and hamster models of CDI. The isolates of epidemic ribotype of *C. difficile* were found to be more virulent in both the murine and hamster models than non-epidemic isolates. In particular, the group of epidemic ribotypes of *C. difficile* had lower LD_50_ values in hamsters. The increased severity of disease was associated with higher levels of Toxin A and Toxin B production found in fecal samples, but not numbers of organisms recovered. The isolates were further characterized for their in vitro virulence phenotypes, e.g. toxin production, growth rates, spore formation and adherence of spores to intestinal epithelial cell lines. Although there were higher levels of toxins produced and greater adherence for the group of epidemic ribotypes, the in vitro profiles of individual isolates were not always predictive of their in vivo virulence.

**Conclusions:**

Overall, the group of epidemic ribotypes of *C. difficile* were more virulent in vivo despite individual isolates having similar phenotypes to the non-epidemic isolates in vitro.

*Clostridioides difficile,* a spore forming bacillus, is the cause of *C. difficile-*associated disease. In the United States of America (US), the occurrence of *C. difficile* infections (CDI) increased by a factor of 400% between 2000 and 2007 [1]. *C. difficile* is estimated to cause 500,000 infections in the US each year that results in 29,000 deaths and associated annual healthcare costs of approximately $3 billion [2, 3]. Clostridial endospores are essential for the environmental transmittance of *C. difficile* in humans and are resistant to a broad variety of physical and chemical treatments [4, 5]. Within the host, *C. difficile* spores germinate into vegetative cells, which enables colonization of the intestinal tract, toxin production, and eventual disease [6, 7]. Stages of disease progression include intestinal inflammation, perforation, toxic megacolon, pseudo-membranous colitis, and death [7, 8]. Mortality associated with CDI is approximately 5% but has been as high as 20% during particular outbreaks [9]. *C. difficile* is capable of producing two different Rho glucosylating exotoxins, TcdA (toxin A) and TcdB (toxin B) [10, 11], which are responsible for the pathology typically associated with CDI [12, 13]. Toxin A and B both produce multiple cytopathic and cytotoxic effects on the targeted cells [10]. These can include disruption of Rho-dependent signaling, disruption of the actin cytoskeleton and of the tight adherence junctions, all causes of increased epithelial permeability which cause the diarrhea associated with *C. difficile* associated disease [10]. *C. difficile* isolates can produce another toxin, binary toxin, which can disrupt normal cytoskeletal function of cells [14]; however, studies have yet to show that binary toxin plays a significant role in disease severity or virulence [15, 16]. Therefore, both *C. difficile* spores and toxins play an important role in disease transmission and pathogenesis, and these virulence determinates have been shown to vary between different *C. difficile* ribotypes [10, 11, 13, 17].

The increase in the number and severity of CDI in the United States is largely attributed to the emergence of the epidemic *C. difficile* clinical isolates, e.g. BI/NAP1/027 (type 027) and ribotype 078 [18, 19]. Interestingly, ribotype 027 is common among healthcare-associated CDI cases, while the type 078 is more commonly associated with community-acquired CDI [19]. Ribotype 027 is responsible for 19 to 22.5% of hospital acquired CDI cases, and most of these cases are significantly associated with increased disease severity, recurrence, and mortality [19–21]. It was recently suggested that one possibility why ribotypes 027 and 078 have become epidemic strains was due to their ability to utilize low concentrations of the sugar trehalose [18]. The increased usage of trehalose as a food additive in both the US and Europe coincides with the emergence of both ribotype 027 and 078 outbreaks. Thus, the ability to utilize this sugar may provide a competitive advantage over other ribotypes, resulting in the increased frequency of infection within a complex host environment [18]. Still, this does not account for the increased frequency of diagnosis of disease associated with infection with epidemic ribotypes, as well as the increased severity of disease associated with them when compared to other non-epidemic ribotypes.

The apparent increased severity of disease due to the epidemic ribotypes of *C. difficile* suggests that these isolates may be more virulent than other ribotypes, and if so, this is likely linked to enhanced expression of virulence determinates, such as spores and toxins A and B [22]. There are limited studies examining in vivo virulence of multiple isolates of the epidemic ribotypes using animal models [23, 24]. However, there are multiple in vitro studies that characterize type 027’s spore and toxin production, but these studies have produced conflicting results. Some in vitro studies indicate that ribotype 027 has increased spore and toxin production [17, 22, 25, 26]. Increased toxin production was highlighted in a study by Warny et al., which found a ribotype 027 isolate expressing 16 times more toxin A and 23 times more toxin B that other ribotype isolates [22]. In contrast, other in vitro studies found that spore production for other ribotype 027 isolates were not significantly different from other ribotypes, and toxin production by ribotype 027 is not as robust as shown in the study by Warny et al. [27, 28]. These studies, as well as other studies, have not definitively compared the in vitro profiles of various *C. difficile* isolates with their ability to cause disease in vivo*,* leading others to speculate that clinical outcomes may be isolate dependent. Thus, it is unclear whether epidemic ribotypes are more virulent than other ribotypes, and whether the in vitro virulence phenotype of an isolate or ribotype is useful in predicting in vivo virulence of individual isolates.

To examine the virulence of epidemic isolates, we initially determined the in vivo virulence of 13 *C. difficile* isolates (7 non-epidemic and 6 epidemic) in two different animal models of CDI. The first animal model that was used in these studies was the murine model of CDI [23]. Being that mice are less susceptible to *C. difficile,* this model is an excellent shedding model and has been used, with some success, as a survival model [23, 29]. Also, due to this decreased sensitivity to *C. difficile*, the mouse model is better suited for determining subtle differences between isolates that pose an issue in more sensitive animal models, such as toxin production over extended periods of time [20]. The second animal model that was used in these studies is the hamster model of CDI. In contrast to mice, hamsters are very sensitive to *C. difficile* and, though there are differences (i.e., the increased sensitivity), closely parallels the characteristics of clinical *C. difficile-*associated disease in humans [20]. This enhanced sensitivity makes the hamster model of CDI a strong choice for survival studies and the subsequent calculation of LD_50_ values for *C. difficile* strains [29–31], whereas the murine model can be useful in dissecting more subtle differences in virulence, such as in vivo toxin production and shedding of organisms other than lethality [20]. By using this approach, we found collectively that the epidemic isolates had increased virulence in both experimental animal models when compared to non-epidemic isolates. In particular, the group of epidemic ribotypes of *C. difficile* had lower LD_50_ values in hamsters. Additionally, we also examined the in vitro production of toxins A and B, growth rates, spore formation and adherence of spores to intestinal epithelial cell lines, and although there was increase production of toxins and adherence for the group of epidemic isolates, the in vitro profiles of individual isolates were not predictive of their in vivo virulence. Overall, the group of epidemic ribotypes of *C. difficile* were more virulent in vivo despite individual isolates having similar phenotypes to the non-epidemic isolates in vitro.

## Results

### Isolates of the epidemic ribotypes of *C. difficile* are more virulent in the murine CDI model when compared to isolates of non-epidemic ribotypes

A mouse CDI model was used to compare the virulence of the non-epidemic and epidemic *C. difficile* isolates in vivo. This is a frequently used model to study colonization, shedding, disease progression, and, in some cases, survival [23, 29]. For this model, the intestinal microbiome of the mice was disrupted with antibiotics and then they were orally inoculated with approximately 1 × 10^6^ *C. difficile* spores. Survival was monitored for the entire study, and feces were sampled each day for 7 days post-infection and every other day thereafter, until the end of the study (Day 12). *C. difficile* CFU and toxin levels in fecal samples were determined.

**Fig. 1:**
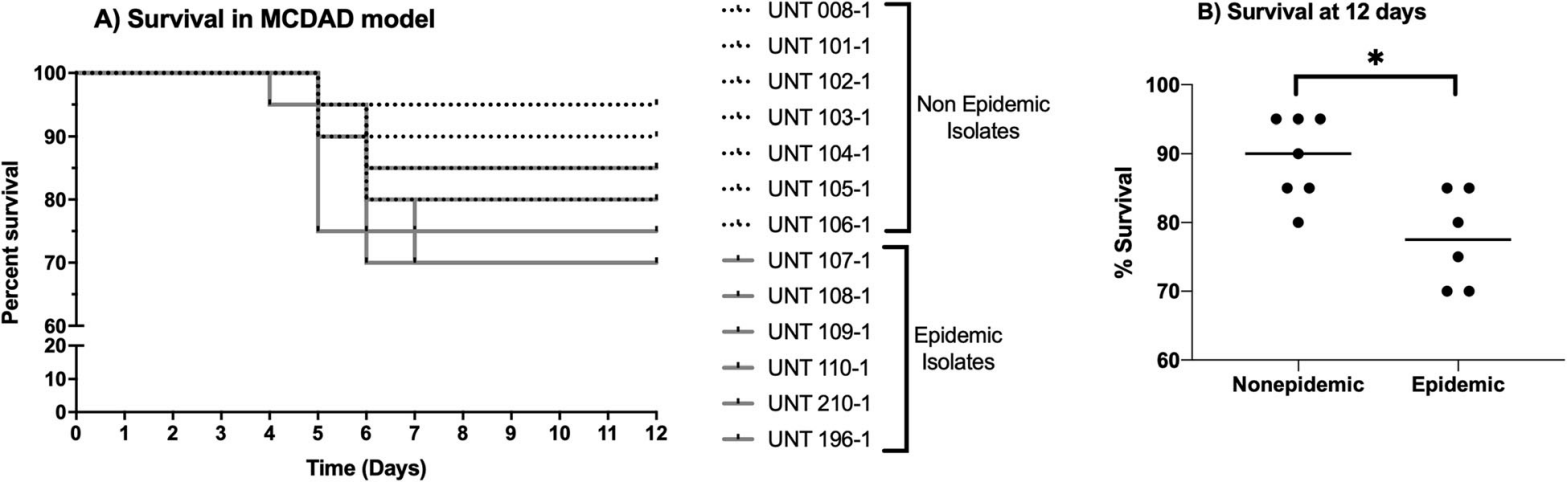
Mice infected with epidemic ribotype isolates had lower survival than mice infected with non-epidemic ribotype isolates. For each isolate, groups (*n* = 20) were housed 5 to a cage and inoculated with approximately 1 × 10^6^ *C. difficile* spores. **a** The non-epidemic ribotype isolates are denoted by black survival curves, and the epidemic ribotypes are denoted by gray. Survival was monitored for 12 days, and there were no additional deaths for any isolate after day 7. **b** Percent survival at 12 days after infection. An asterisk denotes significant difference at *p* ≤ 0.05 (Student’s unpaired t test)

The epidemic ribotype isolates caused greater mortality than those with non-epidemic ribotypes (Fig. 1). The notable exception to this trend was non-epidemic ribotype isolate UNT 106–1. This isolate had a mortality rate that was equivalent to UNT 109–1 and greater than UNT 210–1 (both, epidemic, type 027 isolates). As a whole, mortality rates ranged from 15 to 30% for mice infected with epidemic ribotype isolates, while the mortality rates for nice infected with non-epidemic ribotype isolates ranged from 5 to 20%.

**Fig. 2:**
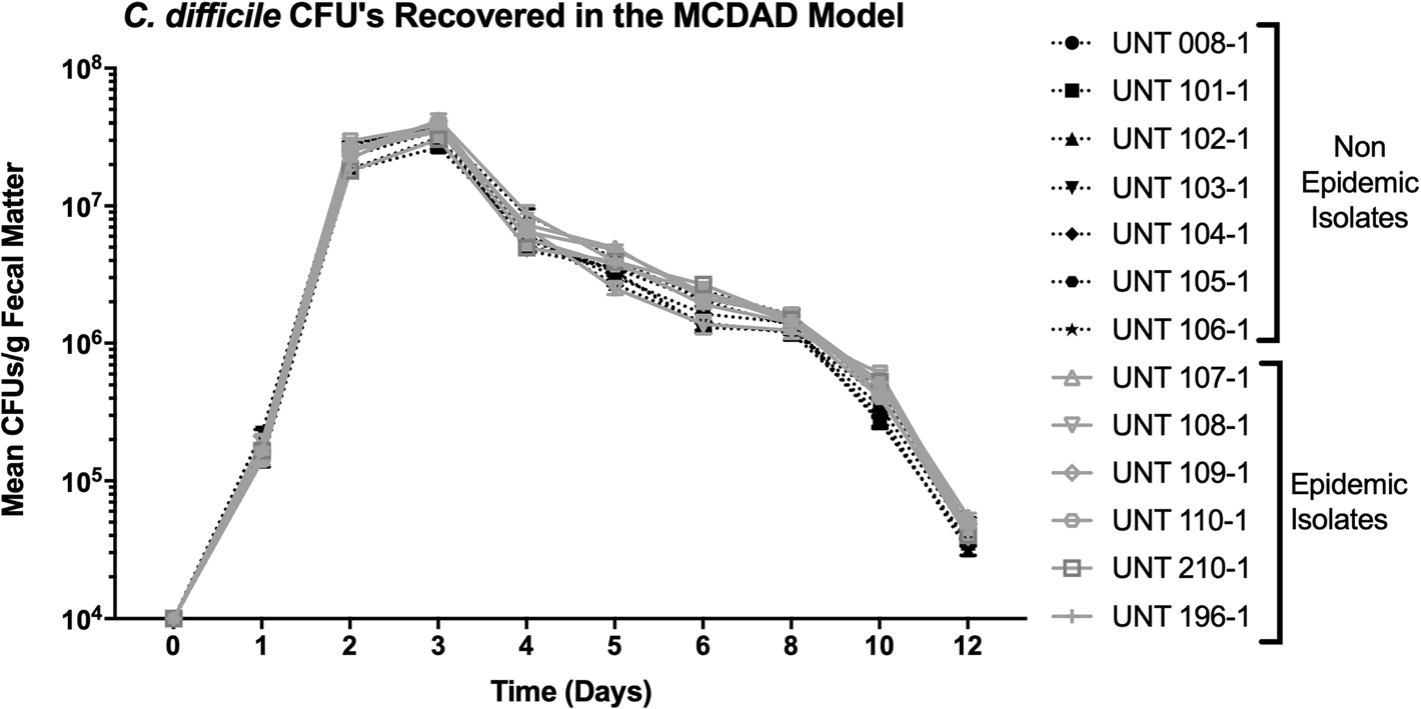
In vivo fecal-associated CFU counts were not different between isolates. For each isolate, groups (*n* = 20) were housed 5 to a cage and inoculated with approximately 1 × 10^6^ *C. difficile* spores. Fecal pellets were then collected, weighed, and processed to measure CFU counts throughout the study. Mean fecal counts were not significantly different between the non-epidemic and epidemic ribotypes, and CFU counts peaked 3 days after infection which declined until the end of the study. These data represent the average of four independent groups, and error bars indicate the standard errors of the means

Despite the differences in survival, there were no significant differences between fecal *C. difficile* CFUs recovered from mice infected with epidemic and nonepidemic ribotype isolates (Fig. 2). All isolates followed a similar pattern of growth, and growth for the isolates reached its apex between 1 × 10^7^ and 1 × 10^8^ CFU per gram of feces on days 2 and 3 of the studies. After this apex, there was a similar decline in the recovered fecal counts observed for each isolate.

**Fig. 3:**
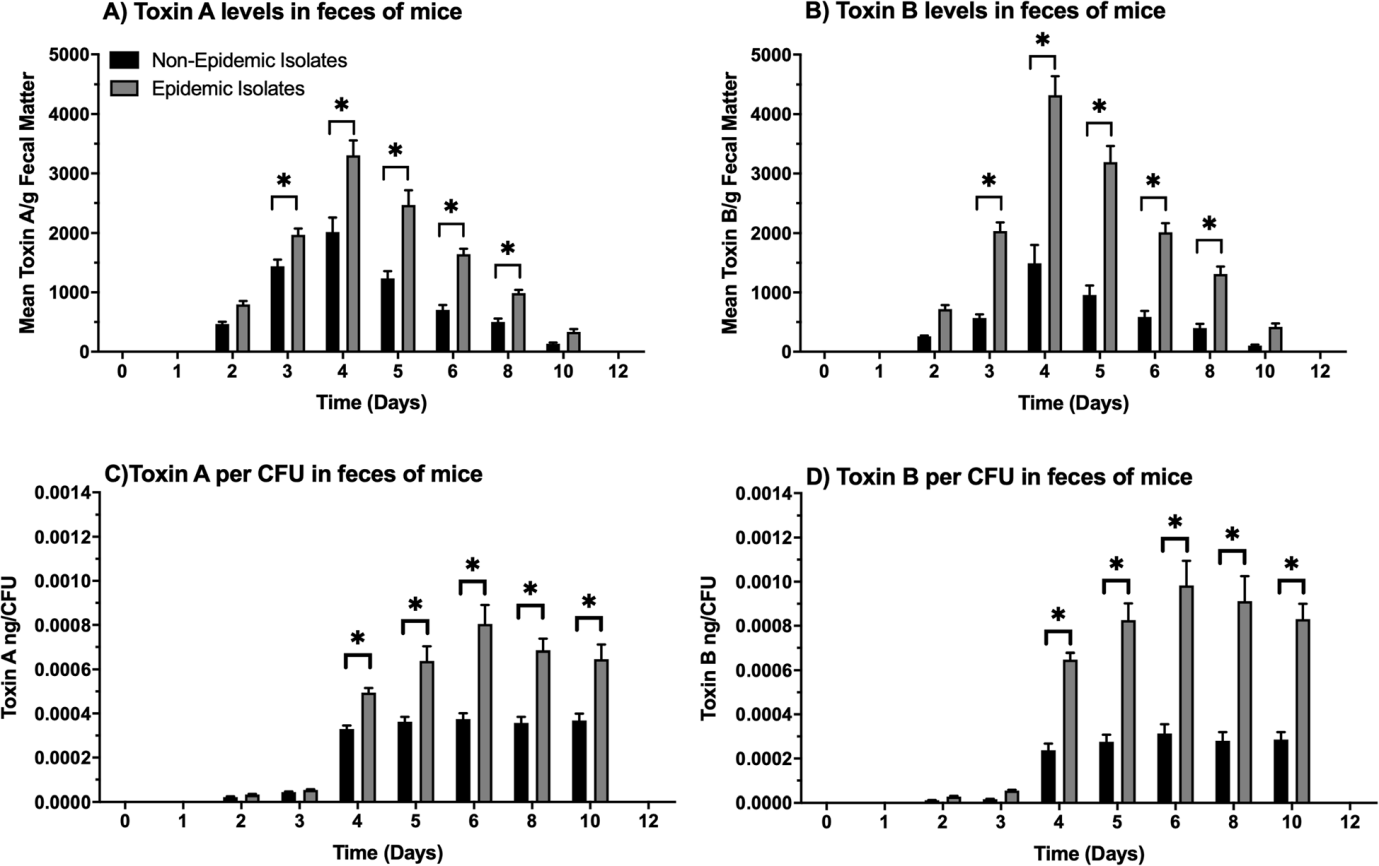
Epidemic ribotype infected mice had significantly more fecal-associated Toxin A and B than mice infected with non-epidemic ribotype isolates of *C. difficile*. For each isolate, groups (*n* = 20) were housed 5 to a cage and inoculated with approximately 1 × 10^6^ *C. difficile* spores. Fecal pellets were then collected, weighed, and processed to measure Toxin A and B concentrations via ELISA. **a** Mean Toxin A titers per gram of feces that was collected from epidemic or non-epidemic ribotype infected mice on days 0 to 12 of the studies. **b** Mean Toxin B titers per gram of feces that was collected from epidemic or non-epidemic ribotype infected mice on days 0 to 12 of the studies. **c** Normalized mean Toxin A titers per CFU that was collected from epidemic or non-epidemic ribotype infected mice on days 0 to 12 of the studies. **d** Normalized mean Toxin B titers per CFU that was collected from epidemic or non-epidemic ribotype infected mice on days 0 to 12 of the studies. These data represent the average of four independent groups, and error bars indicate the standard errors of the means. An asterisk denotes significant difference at *p* ≤ 0.05 (Two-way ANOVA with Tukey’s post-hoc test)

Significant levels of Toxin A and B in fecal samples were found in mice infected with non-epidemic or epidemic ribotype *C. difficile* isolates (Fig. 3). The data per gram of feces were similar to that if normalized to CFU numbers recovered. Measurable concentrations of Toxin A for both the non-epidemic and epidemic ribotype isolates were initially detected 2 days after infection and were continued through day 10 of each study. Toxin A production for both sets of isolates peaked 4 days after infection, and there were significant differences observed between the non-epidemic and epidemic mean Toxins A levels associated with feces collected between days 3–8 (*p* ≤ 0.05). During this time, the feces collected from mice with epidemic ribotype isolates had between 1.5–2.5x higher mean levels of Toxin A/gram than feces collected from mice infected with non-epidemic ribotypes. Similar trends were observed for fecal-associated Toxin B production titers determined for animals infected with epidemic and non-epidemic *C. difficile* ribotype isolates. During this time, between 3-4x higher levels of Toxin B was found in feces collected from epidemic ribotype infected mice than those infected with non-epidemic ribotypes (*p* ≤ 0.05). When toxin levels were normalized with numbers of CFU recovered, Toxin A levels per CFU in feces from epidemic ribotype infected mice were 2-3x more (*p* ≤ 0.05) than feces from mice infected with the non-epidemic ribotypes. In addition, there was approximately 3.3x higher levels of Toxin B per CFU in feces from epidemic ribotype infected mice than the non-epidemic ribotype infected mice. (*p* ≤ 0.05).

### Epidemic ribotype isolates of *C. difficile* are more virulent than non-epidemic ribotype isolates in the hamster model of CDI

The previous studies using the mouse model of CDI suggested the epidemic ribotype isolates were more virulent than the non-epidemic ribotype isolates. The virulence of the two sets of *C. difficile* isolates were further investigated using the hamster model of CDI. The hamster model is well established and shares some common features of *C. difficile* disease associated with the human clinical condition [29, 32]. Like humans, hamsters also exhibit increased susceptibility to *C. difficile* infection after administration of a broad spectrum antibiotic that often leads to consistent clinical disease outcomes in the experimental model [31, 32]. To perform these studies, groups of hamsters were inoculated with a range of spore titers per isolate, and then treated with clindamycin to facilitate infection and subsequent disease establishment. After this, the condition of the hamsters was assessed multiple times a day, and fecal samples were collected daily until the conclusion of the study on day 7. Fecal samples were processed for CFU and assayed for Toxin A and B concentration via ELISA.

**Fig. 4:**
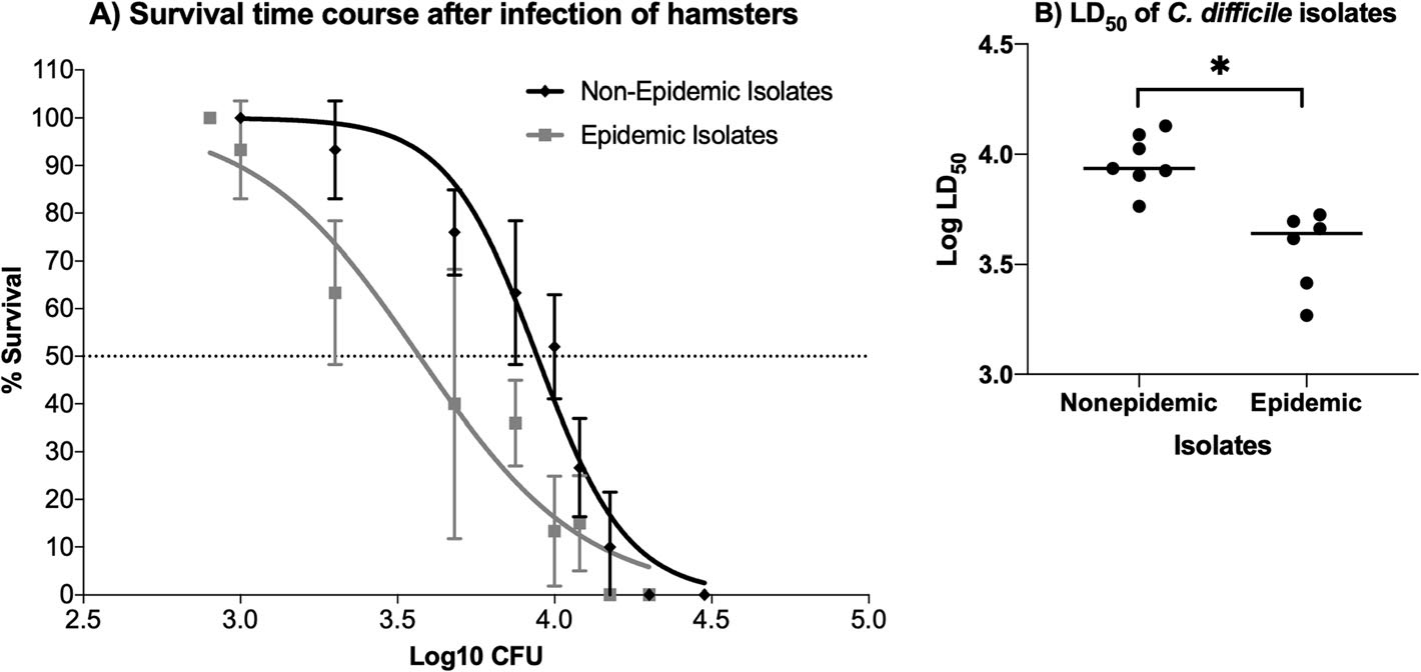
Epidemic ribotype isolates of *C. difficile* are more virulent than non-epidemic isolates in the hamster model of CDI. For each isolate, groups (*n* = 5) were orally inoculated with a titration range of *C. difficile* spores as needed to define the LD_50_. **a** The graph compares the mean survival of each group inoculated with either non-epidemic or epidemic strains at specific log_10_ spore titers. Error bars represent the standard deviation of mean survival percentages at specific spore titers, and average LD_50_ values were calculated for each group with the variable slope model (Y = 100/ (1 + 10^((LogEC_50_ – x) * HillSlope))) and were determined to be significantly different using the extra sum-of-squares F test (*p* < 0.05). **b** The individual LD_50_ values for epidemic and nonepidemic ribotype isolates are shown. An asterisk denotes significant difference at *p* ≤ 0.05 (Student’s unpaired t test)

When LD_50_ values were compared between the isolates in the hamster CDI model, the epidemic isolates had a lower mean LD_50_ value than the non-epidemic isolates did in the model (Fig. 4). The average LD_50_ value was 3.57 ± 0.025 log CFU for hamsters infected with epidemic strains, and hamsters infected with non-epidemic strains had a LD_50_ value of 3.94 ± 0.051 log CFU (*p* ≤ 0.05). As a whole, the LD_50_ values ranged from 3.27–3.72 log CFU for the hamsters infected with epidemic ribotype strains, while the LD_50_ values for the hamsters infected with non-epidemic ribotype isolates ranged from 3.76–4.13 log CFU.

**Fig. 5:**
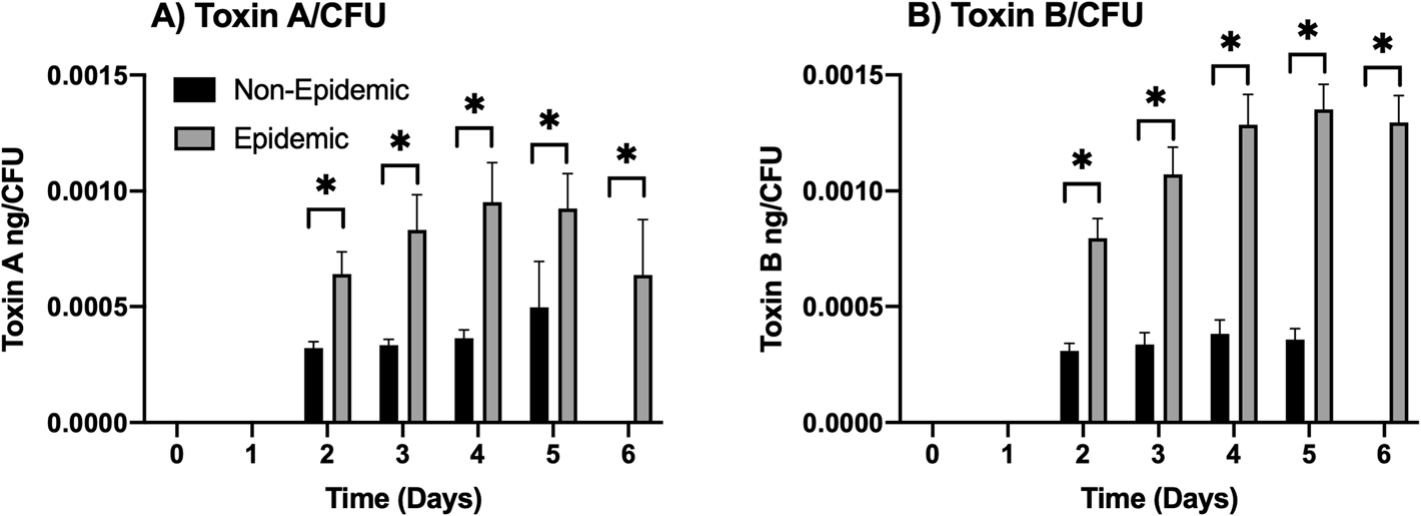
Fecal-associated Toxin A and B was significantly higher in hamsters infected with epidemic ribotype of *C. difficile* in the hamster CDI model. For each isolate, hamsters were split into groups of 5, housed individually, and orally inoculated with a specific titer of spores. Fecal pellets were collected every 24 h, then weighed and processed for detection of Toxin A and B by an ELISA. Toxin levels were normalized to the numbers of CFU recovered. **a** Toxin A and **b** Toxin B levels were higher in hamsters infected with epidemic isolates. These data represent the average of 5 independent data points, and error bars indicated the standard error of the means. Asterisks denote significant differences between toxin values at *p* < 0.05 (Two-way ANOVA with Tukey’s post-hoc test; *p* < 0.05)

For this model, we chose not to compare fecal-associated CFU counts, because determining the LD_50_ values led to varying inoculation doses for each isolate. Due to differences observed between the isolate’s toxin production in the mouse model, we chose to examine fecal-associated Toxin A and B concentrations to determine if this was similar in the hamster model. To do this, toxin levels/CFU was assayed from the fecal samples collected daily for 6 days after infection, and the results were separated into multiple groups for comparison purposes. Fecal-associated Toxin A and B were initially detected 2 days after infection for both the non-epidemic and the epidemic ribotype infected animals (Fig. 5). When comparing non-epidemic and epidemic ribotype infected groups that survived, the epidemic isolate infected hamsters had approximately 2-3x more Toxin A/CFU in their feces than did non-epidemic isolate infected hamsters (*p* ≤ 0.05), and the feces collected from epidemic ribotype infected animals had approximately 3-4x Toxin B/CFU higher levels than hamsters infected with isolates of the non-epidemic ribotype (*p* ≤ 0.05).

### In vitro growth and spore production are similar between non-epidemic and epidemic ribotype isolates of *C. difficile*

**Fig. 6:**
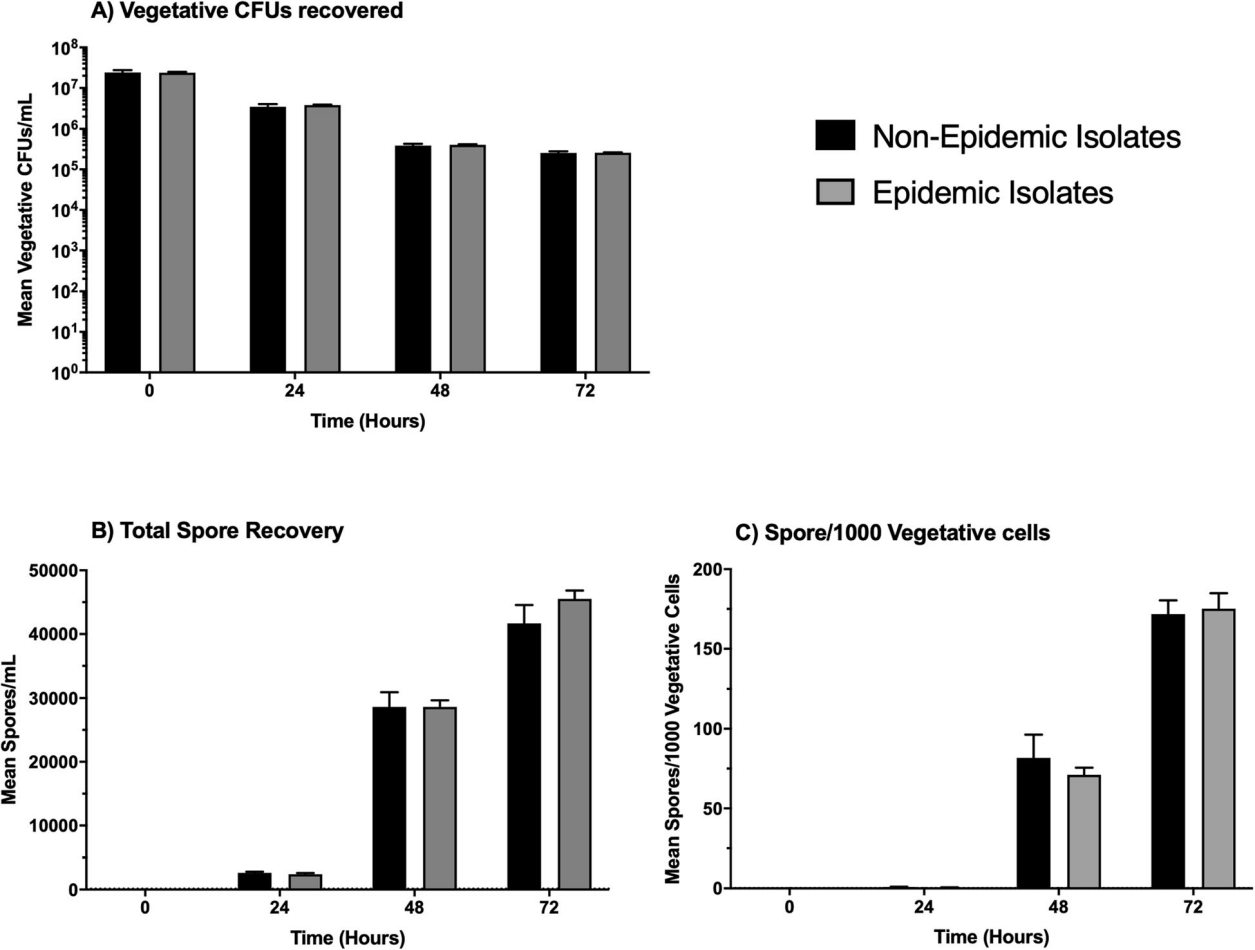
Mean vegetative CFUs and spore recovery between non-epidemic and epidemic ribotype isolates did not differ over 72-h. The 13 isolates (7 non-epidemic and 6 epidemic) were incubated in SM broth over a 72-h period. A representative sample was then taken from each culture and plated on an agar medium ±0.1% taurocholate. The non-epidemic isolates are represented by the black bars, and the epidemic isolates are represented by the gray bars. This data represents the average of three independent experiments and error bars indicate the standard errors of the means. **a** Mean vegetative CFU’s recovered from 72-h SM broth cultures. **b** Mean spores/mL recovered from 72-h SM broth culture. **c** Mean number of spores recovered from SM broth cultures normalized per 1000 vegetative cells recovered at the corresponding time point

Epidemic isolates were shown to be more virulent than non-epidemic isolates in vivo, despite having no differences in recovered CFU. To confirm that there are no inherent differences in growth and spore production of the isolates, in vitro growth and spore formation of all the *C. difficile* isolates were determined over a 72-h period, and, it was found that non-epidemic and epidemic strains exhibited similar in vitro growth patterns. Furthermore, when placed into sporulation medium, there was no difference over a 72-h period between epidemic and non-epidemic isolates in spore formation or the numbers of remaining vegetative cells (Fig. 6, Additional file 1: Figure S1).

### In vitro toxin a and B production is higher in epidemic ribotype isolates than non-epidemic ribotypes

Infection of animals with epidemic ribotype isolates were shown to result in higher levels of Toxin A and Toxin B in fecal samples. Toxin A and Toxin B production is a major factor in intestinal epithelial damage and increased severity of disease [10, 12], and previous studies found variable levels of in vitro toxin production between non-epidemic and epidemic ribotypes [10, 13, 17]. Therefore, we performed sets of in vitro experiments to determine if the non-epidemic and epidemic *C. difficile* isolates produced similar amounts of Toxin A and Toxin B over a 72-h period. These studies were performed in parallel with the sporulation studies, and spent medium from each time point was used to determine Toxin A and B titers by ELISA.

**Fig. 7:**
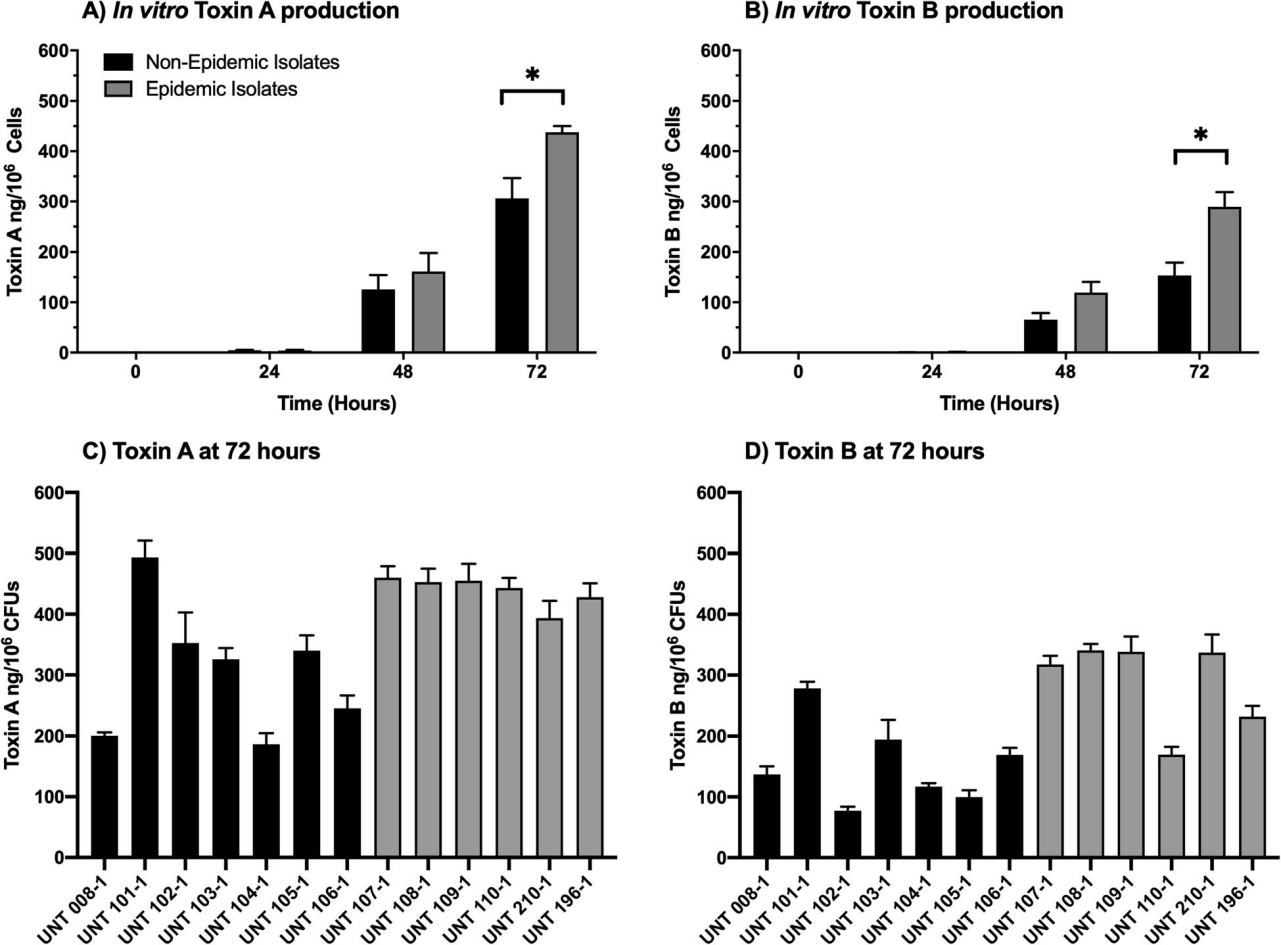
Normalized in vitro Toxin A and B production differs between non-epidemic and epidemic ribotype isolates at 72-h. The 13 isolates (7 non-epidemic and 6 epidemic) were cultured in SM broth over a 72-h period. **a** Toxin A and **b** Toxin B production was determined from spent medium by ELISA and normalized per 10^6^ vegetative cells recovered. **c** Toxin A and **d** levels at 72 h in culture for each of the individual isolates are shown. Mean toxin titers for non-epidemic isolates are represented by the black bars, and mean toxin titers for epidemic isolates are represented by the gray bars. These data represent the average of three independent experiments, and error bars indicate the standard errors of the means. An asterisk denotes significant difference at *p* < 0.05 (Two-way ANOVA with Tukey’s post-hoc test; *p* < 0.05)

Mean Toxin A and B values were significantly different between the non-epidemic and epidemic ribotype groups at 72-h (Fig. 7) (Two-way ANOVA with Tukey’s post-hoc test; *p* < 0.05). Isolates with the epidemic ribotype produced approximately 1.4x Toxin A and 2x Toxin B than the non-epidemic isolates in 72-h culture. Although there was a significant difference between the groups, there was variability within the individual isolates within non-epidemic and epidemic ribotype groups. For example, the non-epidemic isolate UNT 101–1 produced Toxin A levels that were not significantly different than the levels produced by the epidemic isolates, while producing Toxin B levels significantly greater than two epidemic isolates (UNT 110–1 and UNT196–1; *p* ≤ 0.05). Toxin B levels were more variable within the groups of isolates than Toxin A.

### In vitro adherence of non-epidemic and epidemic ribotype *C. difficile* spores to Caco-2 and C2BBe1 cells are significantly different

Adherence to intestinal epithelial cells is thought to be integral for *C. difficile* colonization and subsequent infection. Therefore, in vitro studies comparing the ability of non-epidemic and epidemic spores to adhere to two different intestinal epithelial cell lines (i.e., Caco-2 and C2BBe1) were done. Caco-2 cells are traditionally used for studies involving intestinal epithelial cells, while C2BBe1 cells are a clone of Caco-2 cells [33]. The C2BBe1 cells are more homogenous than Caco-2 cells in regards to brush border expression and are morphologically similar to the human colon [34]. To perform these studies, wells containing confluent intestinal epithelial cells were infected with *C. difficile* spores and incubated for 3-h. Selection of this timepoint was chosen based on preliminary studies, where adhesion was found to plateau at 3-h. Non-adherent spores were removed by washing plates, and intestinal cells were collected and pleated to determine percent adherence.

**Fig. 8:**
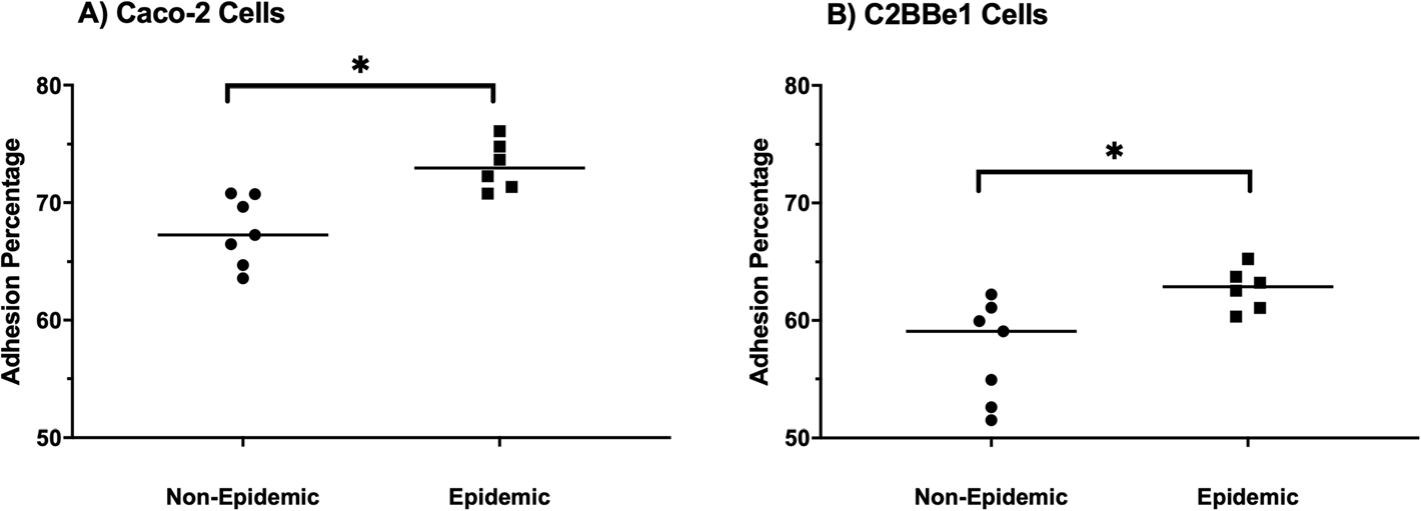
Spores of epidemic ribotype adhere significantly different than those from the non-epidemic ribotype in vitro to Caco-2 and C2BBe1 Cells. *C. difficile* isolates (7 non-epidemic and 6 epidemic) were incubated with either Caco-2 or C2BBe1 cells for 3-h, washed, plated and counted to determine the adhesion for each isolate. The non-epidemic isolates are denoted by the black symbols and the epidemic isolates by the gray symbols. **a** The isolates were incubated with Caco-2 cells and the mean adhesion percentages were determined as the percentage of spores bound after washing as compared to the original inoculum dose. **b** The isolates were incubated with C2BBe1 cells and the mean adhesion percentages were determined as the percentage of the spores bound after washing as compared to the original inoculum dose. These data represent the average of three independent experiments and error bars indicate the standard errors of the means, and a statistically significant difference between each group at *p* < 0.05 (One-way ANOVA with Tukey’s post-hoc test; *p* < 0.05)

Overall, the mean percentages of adhered epidemic *C. difficile* spores to both intestinal epithelial cells were significantly higher than the mean percentages determined for adherent non-epidemic spores. Spores from epidemic isolates adhered at a 5% higher level to Caco-2 cells than non-epidemic isolates (Fig. 8) (*p* ≤ 0.05). When comparing the non-epidemic and epidemic spore’s adherence to C2BBe1 cells, there was also a 5% difference between the groups (*p* ≤ 0.05).

## Discussion

With the identification of the epidemic NAP/BI/027 ribotype, there has been an ongoing debate if this genetic cluster of *C. difficile* is more virulent than non-epidemic ribotypes [8, 11, 19, 20, 22, 25, 35, 36]. This debate is supported by papers which have stated the ribotype 027 is more virulent and relatively more prevalent cause of disease because it hyper-produces toxins and spores in vitro [17, 19, 24, 25]. Whereas, other papers have stated there is little differences between the 027 ribotype and other non-027 ribotypes in vitro [8, 11, 37]. However, there is also a question whether in vitro characterizations accurately predict the in vivo virulence of individual *C. difficile* isolate or a group of isolates of the same ribotype. Therefore, we undertook a comprehensive set of in vitro and in vivo studies of 13 *C. difficile* isolates (7 of non-epidemic ribotypes and 6 of epidemic ribotypes) to examine whether isolates of the epidemic ribotype are more virulent than non-epidemic isolates in vivo. To do this, we not only characterized the isolates in vitro*,* but also used a unique approach of characterizing the same isolates’ in vivo virulence within two different animal models of *C. difficile* infection. Each of the animal models are valuable in understanding various contributing factors of *C. difficile* disease. There are strength and weaknesses of each animal model [29, 32], and using both models decreased the potential skewing of the data associated with the weaknesses and strengths of each model. With this approach, we were able to answer questions about *C. difficile*’s epidemic ribotype in comparison to other non-epidemic ribotypes. Such as, is there truly a difference between non-epidemic and epidemic isolate’s in vivo virulence, and is an isolate’s in vitro virulence phenotype predictive of its in vivo virulence?

As a group, isolates of an epidemic ribotype were more virulent than those from non-epidemic ribotypes, although there was variability within each group of ribotypes. Difference in in vivo virulence was found using two animal models, murine and hamster. The mouse model is an excellent shedding model and has been used, with some success, as a survival model [23, 29]. In mice, there were differences in survival after infection with epidemic isolates or non-epidemic isolates. Between 4 and 8 days after infection the average mortality of the mice infected with epidemic isolates of 22.5% while the mice infected with non-epidemic isolates averaged 10.7% mortality. In the hamster model *C. difficile* infection, we confirmed the results observed in the mouse CDI model in that epidemic isolates have increased virulence when compared to the non-epidemic isolates. Compared to both mice and humans, hamsters are more sensitive to *C. difficile* toxin, and this sensitivity makes it a strong choice as a survival model and determining the median lethal dose or LD_50_ value [29, 32]. Epidemic isolates had significantly lower mean LD_50_ values in the hamster model than the non-epidemic isolates. Our results clearly demonstrate differences in virulence between the groups of epidemic and non-epidemic isolates, but to further examine these difference, future studies to examine the type and extent of tissue damage using histopathology would provide additional insights on differences in disease and mechanisms of virulence, especially in the murine model. Overall, our studies demonstrate that the *C. difficile* strains of the epidemic ribotype were more virulent than non-epidemic isolates in vivo.

The differences in survival in mice infected with epidemic and non-epidemic isolates occurred even though the numbers of *C. difficile* recovered from the animals were the same, suggesting a factor other than growth are responsible for the difference in virulence. Consistent with the in vivo results, there were no differences in the in vitro growth or spore formation between epidemic and non-epidemic isolates. Previous in vitro studies found that epidemic ribotype 027 isolates produced more spores and higher levels of toxin than nonepidemic isolates [17, 35]. Although we did not show a difference in spore formation, there was a significant difference in toxin production between the epidemic isolates and the non-epidemic isolates in the animal models of *C. difficile* infection. In both mice and hamsters, there were two to three times higher levels of both toxins after infection with the epidemic isolates. Consistent with the previous published studies [17, 22], higher levels of toxin production, by epidemic isolates, was also found during in vitro culture, but was only significant at 72-h in culture. Approximately two times more toxin production was associated with the epidemic isolates in in vitro cultures when compared to the non-epidemic isolates. It is worth noting increased toxin production for some ribotype 027 isolates is associated with genetic mutations within its pathogenicity island, this could also play a role in the epidemic isolates’ increased virulence in vivo [25, 38, 39]. Thus, the increased virulence of the epidemic isolates was linked to the higher production of Toxin A and Toxin B.

Although toxin levels may be the most critical factor involved in increased disease severity, there may be other factors. For example, one factor that is speculated to contribute to *C. difficile* virulence is an isolate’s ability to adhere to intestinal epithelium, but although it is accepted that adherence is an important step for other pathogens, it is currently not clear what the significance is of adherence for this *C. difficile* in clinical disease. Studies do suggest it may play a role. Adherence of *C. difficile* spores to epithelium is dependent on the characteristics of exosporium, and the composition of this outmost layer can vary between strains [40–42]. Recently, two cysteine-rich proteins, *cdeC* and *cdeM*, were shown to influence the ability of *C. difficile* spores to adhere to intestinal epithelium [40]. In the mouse model of infection, spores lacking the CdeC protein had increased colonization rates, recurrence rate, and were correlated with higher toxin titers during disease [40]. These results suggest that adherence mediated factors could play a role in the increased virulence associated with the epidemic isolates. In the current studies, the ability of *C. difficile* spores to adhere to two sets of human epithelial cells, Caco-2 and C2BBe1, in vitro was investigated, and the epidemic isolates had about 5% greater adherence to both cell lines than non-epidemic isolates. The ability of the epidemic strains to better bind to the epithelium suggests that these strains will more easily reach the inoculation threshold needed for the establishment of disease. In addition to adherence mediated factors, the spore coat also harbors varying receptors for germination which respond to germanites and co-germinates [43]. Work by Carlson et al. has shown that epidemic isolates respond to more optimized conditions for germinations, and, in turn, this led to more severe disease due to these ribotypes [43]. Though the exact reasons for this has not been elucidated, it is hypothesized that more efficient germination could lead to lower inoculation doses of spores needed to cause disease [43]. In support, lower doses of epidemic ribotype isolates are needed to cause disease, e.g. LD_50,_ in the hamster, but further studies are needed.

In vitro virulence phenotypes of individual *C. difficile* isolates were not predictive of their in vivo virulence. Although the group of epidemic isolates had higher levels of toxin production in vitro, the level of toxin production in vitro did not predict in vivo virulence for each individual isolate. For example, UNT 101–1, a non-epidemic isolate, expressed Toxin A and Toxin B at levels similar to those of the epidemic isolates in in vitro cultures. In contrast, in vitro characterizations showed that UNT 110–1 and 210–1, two epidemic isolates, had toxin levels that were approximately equal with non-epidemic isolates. However, UNT 101–1, though producing high levels of toxin in vitro, was one of the least virulent isolates in vivo, while UNT 110–1 and 210–1 were equal to other epidemic isolates’ observed virulence in the mouse and hamster CDI models. Not only does this suggest that the evaluation of an individual isolate’s virulence should be done using an in vivo model, but it is a strong possibility that factors in the in vivo environment influence an isolate’s toxin production and virulence [40, 44, 45]. In fact, previous studies demonstrate that *C. difficile* epidemic ribotype isolates can have increased in vivo fitness compared to non-epidemic isolates [18, 24]. They are capable of interacting more efficiently with metabolites produced by the host’s GI microbiome and have the ability to utilize additional nutrients that other ribotypes are unable to use. In addition, other factors may contribute to the in vivo virulence of *C. difficile*. For example, although the role of binary toxin in virulence is unclear [15, 16], a study suggests that binary toxin may suppress host immune responses which results in enhanced virulence of epidemic ribotype 027 strains in a mouse model [46]. Most likely complex combinations of factors of *C. difficile* influences the outcome of infection, and to further complicate the ability to assess virulence solely using in vitro studies, the level and types of factors may be differentially expressed in the in vivo environment. Thus, in vitro characterization of virulence factors produced by *C. difficile* alone is not reliable approach to assess the potential to cause disease by individual isolates, but this approach may still be useful in comparing the potential of different groups, e.g. ribotypes, of organisms to cause disease.

Overall, these studies demonstrated that epidemic ribotypes of *C. difficile* are likely to be more virulent than non-epidemic ribotypes. Within the last 10 years, *C. difficile* has become an ever-increasing threat, even being designated an urgent threat level organism in 2013 by the Centers for Disease Control, and the major reason for this is linked to the rise of the epidemic NAP/BI/027 ribotype, along with other “hyper-virulent” ribotypes [19, 26]. Results described in these studies provide a comprehensive examination of virulence between different *C. difficile* isolates through multiple methods and provides an important contribution in further understanding what causes the NAP/BI/027 ribotype to be labelled as, epidemic, hyper-virulent, and such a prevalent threat to healthcare. Previous studies debated whether the current epidemic ribotypes are more virulent than the non-epidemic ribotypes [11, 17, 19, 23, 25, 35]. This appears to be the first study to compare the abilities of isolates of epidemic and non-epidemic ribotypes to cause disease in both the mice and hamster models of CDI. Although all *C. difficile* isolates examined were able to cause disease in both hamsters and mice, the group of isolates with epidemic ribotype caused more severe disease than the non-epidemic group of isolates, providing a compelling case that the epidemic ribotype is indeed more virulent. Additionally, the in vivo and in vitro data supports the idea that the levels of toxins A and B production are likely to contribute to the increased virulence of the epidemic isolates. Other factors, such as the ability to adhere to epithelial cells, may also play a role. However, there was variability in disease severity between individual isolates within the group of epidemic and non-epidemic ribotypes, with one non-epidemic isolate caused disease as severe as one of the epidemic strains. Furthermore, in vitro expression of virulence factors, such as toxin production and adherence to epithelial cells, corresponded with disease potential of the ribotype groups, but was not a reliable approach to assess the potential to cause disease by individual isolates. These results suggest a link between the ability to cause disease and the likelihood of a ribotype’s ability to be epidemic and more easily transmissible between hosts. However, further studies are needed to directly link the ribotype with increased virulence and spread of infection.

## Methods

### Bacterial strains and Ribotype confirmation

All *C. difficile* isolates used in this study are listed in Table 1. *C. difficile* UNT 101–1 to UNT-110-1 were kindly provided by Dr. Curtis Donskey (Cleveland VA); UNT 008–1, UNT 210–1, and UNT 196–1 were obtained from the American Type Culture Collection (ATCC). The source of relevant characteristics of each isolate can be found in Table 1. Ribotypes were confirmed by running polymerase chain reaction (PCR) ribotyping with primers found in Bidet *et. al.* [47]. PCR fragments were analyzed in a Hitachi 3500xL genetic analyzer with a 36 cm capillary loaded with a POP4 gel (Applied Biosystems). The size of each peak was determined using Peak Scanner software (Applied Biosystems). A database was generated from the results of the capillary gel electrophoresis-based PCR ribotyping result of each strain (http://webribo.ages.at). An error margin of ±4 bp was incorporated into the analysis algorithm of the database [48].

**Table 1:** *Clostridioides difficile* Strain Designation, Sources, and Characteristics. This table denotes the source of the individual isolates, other designations for each isolate, and some of the major characteristics associated with each of the isolates

*C. difficile* Isolates and Sources		
UNT Strain #	Source	Relevant Characteristics
UNT 101–1	Ohio VA Medical Center (Curtis Donskey)	Non-epidemic (Ribotype 014/0), Other Designation VA1
UNT 102–1	Ohio VA Medical Center (Curtis Donskey)	Non-epidemic (Ribotype 660), Other Designation VA10
UNT 103–1	Ohio VA Medical Center (Curtis Donskey)	Non-epidemic (Ribotype 428), REA J-type strain, binary toxin negative, non-epidemic, Other Designation VA 11
UNT 104–1	Ohio VA Medical Center (Curtis Donskey)	Non-epidemic (Ribotype 428), Other Designation UH15
UNT 105–1	Ohio VA Medical Center (Curtis Donskey)	Non-epidemic (Ribotype 053), Other Designation UH18
UNT 106–1	Ohio VA Medical Center (Curtis Donskey)	Epidemic (BI/NAP1, binary toxin positive, Ribotype 027), Other Designation VA5
UNT 107–1	Ohio VA Medical Center (Curtis Donskey)	Epidemic (BI/NAP1, binary toxin positive, Ribotype 027), Other Designation VA17
UNT 108–1	Ohio VA Medical Center (Curtis Donskey)	Epidemic (BI/NAP1, binary toxin positive, Ribotype 027), Other Designation VA20
UNT 109–1	Ohio VA Medical Center (Curtis Donskey)	Epidemic (BI/NAP1, binary toxin positive, Ribotype 027), Other Designation CC20
UNT 110–1	Ohio VA Medical Center (Curtis Donskey)	NAP-1, Epidemic, Other Designation L32
UNT 196–1	ATCC	Epidemic (Ribotype 078), BAA-1875 (Other Designation: 5325), Binary toxin positive, Toxinotype V PFGE tye NAP7, REA type BI 8
UNT 210–1	ATCC	Epidemic (Ribotype 027) BAA-1870; Binary toxin positive, Toxinotype IIIb PFGE tye NAP1, REA type BI 8
UNT 008–1	ATCC	Non-epidemic (Ribotype 012), BAA-1382

### Media

Sporulation medium (SM) contained 90 g Trypticase Peptone, 5 g Proteose Peptone no. 3, 1 g Ammonium Sulfate, and 1.5 g of Tris in 1 l of distilled water. The pH was adjusted to 7.4 at 37^o^ with 1 M NaOH. SM is a broth medium made according to what has been previously described [49].

TSA with 5% blood agar was made with 1 L of distilled water (DI), 30 g of TSB, and 15 g of granulated agar with constant mixing over low heat. Once the granulated agar was dissolved, the mixture was autoclaved (20 min, 121 °C, 15 psi). Once cooled to approximately 50 °C, 50 mL of the medium was removed, and 50 mL of sterile defibrinated sheep blood (Remel, Lenexa, KS) was added and mixed into the medium. Approximately 12 mL of medium was then poured into petri dishes and cooled overnight to solidify and stored in a 4 °C refrigerator until used.

TGY-vegetative medium contained 5 g Tryptone, 5 g Yeast extract, 1 g Glucose, 1 g Potassium Phosphate, 15 g agar, and 1 l of distilled water. This liquid-based medium was made according to what has been previously published [50].

Columbia horse blood agar with 0.1% sodium taurocholate was made by adding 869 mL of distilled water, in combination with 35 g of Columbia broth (Remel), and 15 g of Difco Agar, granulated (BD). The mixture was autoclaved (20 min, 121 °C, 15 psi). Once cooled, 70 mL of horse blood and 50 mL of a 20 mg/mL stock of sodium taurocholate, 10 mL of a 50 mg/Ml stock of cycloserine and 1 mL of a 15.5 mg/mL stock of cefoxitin were also added.

### Preparation of *C. difficile* spore stocks

Spore stocks of each *C. difficile* strain were generated for use in the cellular adherence assay and the experimental animal models of CDI. These stocks were generated by growing each strain on 5% TSAb plates incubated at 37 °C in anaerobic conditions for 7 days. Plate growth was collected in a 1X PBS solution containing 1% (V/V) Tween-80 (ST-80), and suspensions were washed 3 times in equal volumes of ST-80. Suspensions were incubated for 1 h at 65 ± 2 °C, washed with ST-80, and re-suspended in 4 mL of sterile nanopore water. Suspensions were then stored overnight at 4 °C in order to promote the maturation of endospores for each strain. Spores were separated from vegetative cells and residual debris by density gradient centrifugation (10 min at 4500 x *g*) with a 25% (W/V) HistoDenz solution. Spore pellets were washed 3 times with ST-80 and suspended in sterile nanopore water to a final volume of 2 mL. Spore stocks for each strain were stored at − 80 °C until used in in vitro or in vivo studies, and the numbers of organisms given for infection or used in in vitro studies were confirmed for each study.

### Mouse *C. difficile* associated disease model

Female C57 BL/6 mice that were 7 to 8 weeks old were obtained from Charles River Laboratory and housed in sterile caging for the in-life portion of each study. Animals were randomly organized into groups of 20 (*n* = 20) and placed on drinking water supplemented with a cocktail of antibiotics immediately upon arrival. These antibiotics and their concentrations were: Kanamycin (0.4 mg/mL), Colistin (850 units/mL), Gentamicin (0.035 mg/mL), Metronidazole (.215 mg/mL), Vancomycin (0.045 mg/mL) [23]. Animals were left on the antibiotic supplemented water for 5 days, and then switched to normal water for 24 h. Mice were orally inoculated with 1 × 10^6^ *C. difficile* spores, and clindamycin was administered subcutaneously at 10 mg/kg of body weight. Starting the day of infection, and each day after, approximately 0.1–0.2 g of feces was collected from cages to determine *C. difficile* counts and associated amounts of toxin A and B. Bedding was changed daily to ensure fresh feces were collected for analysis, and census of survivors were recorded daily for 14 days after infection. Feces were weighed before sterile 1x PBS was added to the recovered feces, this solution was then homogenized, and 1 mL was separated for each total CFU recovery, spore recovery, and toxin A and B expression. Viable cell counts, spore counts, and toxin expression were quantified as described in the Material and Methods. The homogenized solution separated for spore quantification was heated to 65 ± 2 °C for 1 h to facilitate the isolation of only spores, while the fecal matter separated for toxin expression was diluted approximately 100x - 500x for quantification. This allowed it to fall within detection range of the ELISA used to determine toxin concentration.

### Hamster LD-50/Survival *C. difficile* associated disease models

Male Golden Syrian hamsters that were 6 to 7 weeks old were purchased from Envigo RMS Inc., and individually housed in sterile cages. Up to 30 hamsters were used in each study with 5 animals in each group that were orally inoculated with a designated spore titer of each strain. The animals were inoculated with 0.5 mL of *C. difficile* spores from a spore preparation culture though oral gavage. The inoculation dose for all strains ranged from 800 to 30,000 spores/mL, and the exact titers chosen for each strain were based on previously conducted studies and observation of higher titers with non-epidemic and epidemic strains. Clindamycin was administered subcutaneously to each animal at 10 mg/kg per body weight approximately 24 h after infection. Starting the day of infection, and each day after, approximately 0.1 to 0.2 g of feces was collected individually from each cage to determine *C. difficile* counts and associated amounts of toxin A and B. Bedding was changed daily to ensure fresh feces were collected for analysis, and census of survivors were recorded daily for 7 days after infection. Cecal fluid was collected from deceased hamsters for *C. difficile* enumeration and toxin A and B quantification. Feces were weighed before sterile 1x PBS was added to the recovered feces, this solution was then homogenized, and 1 mL was separated for each total CFU recovery, spore recovery, and toxin A and B expression. Viable cell counts, spore counts, and toxin expression were quantified as described in the Material and Methods. The homogenized solution separated for spore quantification was heated to 65 ± 2 °C for 1 h to facilitate the isolation of only spores, and the fecal matter separated for toxin expression was diluted approximately 100x - 500x for quantification. Cecal fluid was processed identically to the fecal samples, with the exception that they were not homogenized. This allowed it to fall within detection range of the ELISA used to determine toxin concentration.

### In vitro growth of *C. difficile* vegetative cells and spore formation

Plate growth of each *C. difficile* isolate was transferred into TGY-veg broth and anaerobically incubated at 37 °C for 24 h. TGY-veg associated growth for each strain was adjusted to an optical density of 0.1 (600 nm) in either SM or TGY-veg broth, which were anaerobically incubated at 37 °C. Samples from each broth culture were collected in triplicate every 24 h through 72 h of total incubation, and these samples were 10-fold serially diluted and plated onto Columbia horse blood agar. Additionally, a second sample from each culture were possessed for spore counts by incubating each sample in an equal volume of 200 proof ethanol for 30 min, and then incubating the samples at 65 ± 2 °C for 1 h. The ethanol and heat-treated samples were centrifuged, washed with PBS, and the spore-containing pellets were suspended in a volume of PBS equal to the original volume of the sample. Ethanol and heat-treatment at 65 ± 2 °C were tested and sufficient to remove all viable vegetative cells during this stage. The spore suspension of each sample was 10-fold serially diluted and plated on Columbia horse blood agar supplemented with 0.1% sodium taurocholate. Both sets of plates were anaerobically incubated at 37 °C for 48 h and colony counts were used to calculate the vegetative CFU or spore counts per mL at each time point.

In addition to determining spore counts associated with each culture by counting the colonies recovered on agar media, the Schaeffer-Fulton endospore staining method was used to visually enumerate spores associated in 72-h cultures of each *C. difficile* isolate. This was done by generating heat-fixed smears of samples taken from each culture every 24 h on glass slides and staining with 0.5% (W/V) malachite green as each slide was being steamed for 5 min. Slides were counterstained with Gram’s safranin for 2 min in order to contrast vegetative cells from endospores and spores in each sample. The number of endospores and free spores were visually counted among 100 non-sporulating vegetative cells with a bright-field microscope at 1000x total magnification, and the percentage of cells that had undergone sporulation was calculated for each *C. difficile* strain in triplicate at each 24-h time point.

At the time of the viable cell quantification, 1.0 mL from the same sample vials were pipetted into 1.5 mL centrifuge tubes and centrifuged at 10,000 x g for 5 min. The supernatant was pipetted into a new 1.5 mL centrifuge tube and stored at − 80 °C until the quantification was performed.

### Quantification of toxins

The levels of toxins A (TcdA) and B (TcdB) in fecal and culture samples were determined using an enzyme-linked immunosorbent assay kit purchased from tgcBIOMICS (Bingen, Germany). Samples were centrifuged at 10,000 *x g* for 5 min, and the recovered supernatants were diluted in kit supplied sample buffer. Toxin A and B concentration values for each sample were interpolated from standard curves generated for each toxin by non-linear regression analysis.

### In vitro *C. difficile* adhesion assay

The Caco-2 cell line (ATCC HTB-37) and the C2BBe1 cell line were purchased from the ATCC. The Caco-2 cells were cultured in Eagles Minimal Essential Medium (EMEM) supplemented with 20% (V/V) fetal bovine serum (FBS), which was heat-inactivated, and 2 mM L-glutamine. The C2BBe1 cells were cultured in Dulbecco’s Modified Eagle’s Medium (DMEM) supplemented with 0.01 mg/mL human transferrin and 10% (V/V) FBS. Other than the use of different growth media, the cell lines were grown and treated the same during the studies. The cells were grown at 37 °C in an atmosphere of 5% CO_2_/95% O_2_**,** and spent media was replaced every other day until the cells reached 80–90% confluency. Caco-2 or C2BBe monolayers were removed from the growth flask with trypsin and transferred into 12-well tissue culture plates, which were placed into an incubator for 2 days, 37 °C in 5% CO_2_/95% O_2,_ to allow the cells to adhere to the wells.

To prepare for the assay, four aliquots of prepared *C. difficile* spore suspension of were washed twice by centrifugation and resuspended in PBS. For the adhesion assay, non-supplemented EMEM or DMEM replaced the medium currently in the wells containing the Caco-2 and C2BBe1 cells at least 1 h prior to the assay, and *C. difficile* spores were seeded at a concentration of roughly 5 × 10^3^ spores per well in triplicate. A negative control with PBS containing no bacteria was also added to additional wells in triplicate. Plates were incubated at 37 °C in 5% CO_2_/95% O_2_ for 3 h. Plates were removed from the incubator and the wells were washed twice with 1x PBS then the Caco-2 cell monolayer was detached from each well by adding a 1% (W/V) trypsin solution and anaerobically incubating the plates for 5 min at 37 °C. The wells were, again, washed with 1x PBS, and the effluent was centrifuged at 8000 x *g* for 5 min. Supernatants were discarded and each pellet suspended in 1 mL of 1x PBS that was ten-fold serially diluted and plated onto Columbia horse blood agar. To enumerate spores the solution was plated on Columbia horse blood agar containing 0.1% sodium taurocholate.

### Statistical analyses

Data were evaluated by One- or Two-way ANOVA with Tukey’s post-hoc test or unpaired Student’s t test. A *p* value ≤0.05 was considered statistically significant. Representation of survival rate against Log10 [daily dose]. LD50 values were calculated with the variable slope model (Y = 100/ (1 + 10 ^((LogEC50 – x) * HillSlope))^) (Curve fitting, Prism 8, Graphpad Software, La Jolla, CA) and were compared for statistical significance using the extra sum-of-squares F test (*p* ≤ 0.05). Analyses were performed using Prism 8 software (Graphpad Software).

## Supplementary information

**Additional file 1.** Supplementary data.

## Data Availability

The datasets generated and analyzed during the current study are available in the Dryad repository (10.5061/dryad.jdfn2z36v).
