# Supplementary figures and images for: Epidemic ribotypes of Clostridium (now Clostridioides) difficile are likely to be more virulent than non-epidemic ribotypes in animal models
Source: BMC Microbiol. 2020 Feb 5;20:27. doi: 10.1186/s12866-020-1710-5 (PMC7003423; doi:10.1186/s12866-020-1710-5)

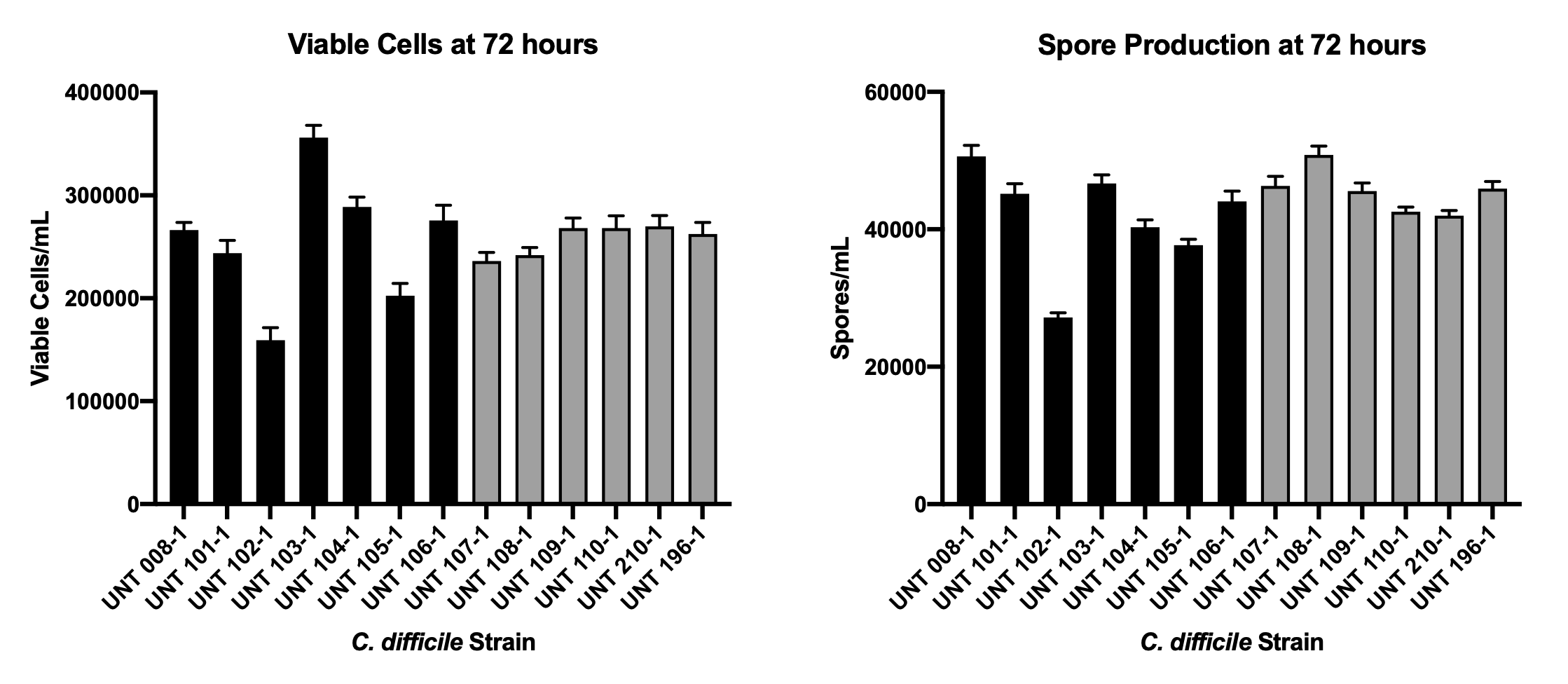

Supplement: Supplementary file 1 — Additional file 1. Supplementary data. [file 12866_2020_1710_MOESM1_ESM.tiff]
